# 1520. Mind the Clap: Reported Disseminated Gonococcal Infections in the United States, 2020–2022

**DOI:** 10.1093/ofid/ofac492.082

**Published:** 2022-12-15

**Authors:** Laura A Quilter, Eric C Tang, Kelly A Johnson, Kathleen Jacobson, Alison Ridpath, Stephanie Cohen, Shobita Rajagopalan, Monica D Munoz, Justin L Holderman, James B Kent, Laura H Bachmann, Kyle Bernstein

**Affiliations:** Centers for Disease Control and Prevention, Atlanta, GA; Sexually Transmitted Diseases Control Branch, Richmond, California; California Department of Public Health & University of California San Francisco, San Francisco, California; CDPH, Richmond, California; Centers for Disease Control and Prevention, Atlanta, GA; San Francisco Department of Public Health, San Francisco, California; Division of HIV and STD Programs, Los Angeles, California; Division of HIV & STD Programs(DHSP), Los Angeles, California; Centers for Disease Control and Prevention, Atlanta, GA; Michigan Department Health and Human Services, Lansing, Michigan; Centers for Disease Control and Prevention, Atlanta, GA; CDC, Atlanta, Georgia

## Abstract

**Background:**

Disseminated gonococcal infections (DGI) are estimated to occur in 0.5% to 3% of untreated *Neisseria gonorrhoeae* (GC) infections; however, surveillance and population-based studies to estimate DGI burden and describe recent DGI epidemiology and clinical manifestations are limited. In response to increased reports of DGI cases and clusters in the U.S., the Centers for Disease Control and Prevention (CDC) developed a surveillance system in 2020. We analyzed data captured by the initial two years of surveillance.

**Methods:**

During 1/1/2020–3/31/2022, U.S. state/local health jurisdictions reported DGI cases to CDC utilizing a standardized case report form that collects demographic, sociobehavioral, and clinical information. A confirmed case was defined as isolation or detection of GC from a disseminated site of infection by culture or nucleic acid amplification test (NAAT); a probable case was defined as clinical manifestations of DGI and isolation or detection of GC from a mucosal site by culture or NAAT.

**Results:**

In total, 274 DGI cases were reported to CDC from 13 U.S. states (65.7% from California): 215 (78.5%) confirmed cases and 59 (21.5%) probable cases. Among DGI cases, 51.1% were cisgender male, 33.2% were ≥ 45 years old, and 26.6% reported methamphetamine use in the past 12 months. Most patients (87.6%) did not have documented underlying immunosuppression or predisposing medical conditions. Among patients with DGI, 85.8% were hospitalized, 41.2% underwent related surgeries, and 2.2% died. Among confirmed cases, the most common disseminated sites of infection were synovial fluid (50.2%) and blood (45.1%). Among 184 (67.2%) patients with DGI who were tested for GC at a mucosal site, 115 (62.5%) were diagnosed with a mucosal GC infection (69.6% urogenital, 27.0% pharyngeal, 12.2% rectal); however, 85.5% of those not diagnosed with mucosal GC were only tested at urogenital sites. Only 89 patients (32.5%) reported mucosal symptoms present at the time of or within the month prior to DGI presentation.

**Conclusion:**

Health care providers should maintain a high degree of suspicion for patients presenting with DGI symptoms given the potential for significant associated morbidity and the low proportion of patients who present with concurrent GC mucosal symptoms.

**Disclosures:**

**Alison Ridpath, MD, MPH**, 3M company: Stocks/Bonds|Abbott Laboratories: Stocks/Bonds|Abbvie Inc.: Stocks/Bonds|Allogene Theraputics: Stocks/Bonds|Amarin Corperation PLC: Stocks/Bonds|infinity Pharmaceutical Companies: Stocks/Bonds|IQVIA Holdings Inc: Stocks/Bonds|Johnson and Johnson: Stocks/Bonds|Medtronic: Stocks/Bonds|pfizer: Stocks/Bonds|Thermo Fisher Scientific: Stocks/Bonds.

